# TikTok for language teachers: Affordances of TikTok on teachers' identity and emotional vulnerability

**DOI:** 10.1016/j.heliyon.2024.e34388

**Published:** 2024-07-11

**Authors:** Henry E. Lemana II, Mark B. Ulla, Lucas Kohnke

**Affiliations:** a Walailak University, Thailand; b The Education University of Hong Kong, Hong Kong

**Keywords:** Emotional vulnerability, Professional identity, Teachers on TikTok, TikTok, Teacher identity

## Abstract

This qualitative study examines how five secondary school TESOL teachers in Thailand use TikTok to express their emotions and shape their professional identities. The research uses semi-structured interviews and thematic analysis to explore the platform's role as an emotional outlet and a medium for enhancing teacher-student relationships. Findings indicate that TikTok helps teachers manage work-related stress and allows them to present authentic selves, facilitating deeper connections with students. This interaction further informs their pedagogical practices and supports their professional identity development. The study suggests that TikTok's ability to bridge emotional expression with professional engagement offers valuable insights for integrating social media into educational frameworks. Recommendations are provided for leveraging these platforms to enhance teachers' well-being and instructional strategies, highlighting the need for further research with a broader sample. This study demonstrates the potential of social media to impact teaching dynamics and professional growth.

## 1 Introduction

Teacher professional identity (TPI) is described as the complex interplay of roles, beliefs, values, attitudes, and practices that define how an individual perceives themselves within the teaching context ([1]) [2]. (2016) have stated that it is “a reflection of the characteristics of the learners and the context of instruction at the level of the classroom, the school, the district, and higher levels of context as these impact on the teacher's aspirations and daily practice” (p. 3). TPI represents a manifestation of self-awareness ([[3], [4], [5]]) that changes when teachers interpret their experiences, engage in self-reflection, exercise agency, and adjust to the changing contours of education (e.g., Ref. [1]).

In the field of Teaching English to Speakers of Other Languages (TESOL), the discussion has expanded to include teacher emotions as a lens to explore the construction and negotiation of TPI (e.g., Refs. [6,7]). For instance Ref. [8], (2016, 2022) has argued that teachers experience emotional struggles in the classroom, which shape their perspectives on their professions, identity, and competence, ultimately impacting their practices and learners. Therefore, emotions provide an avenue for unpacking and understanding teachers' complex professional practices and identity construction ([9]). According to Ref. [7] (2018), “the emotional aspects of teaching can manifest themselves in teachers' feelings about their students, the teaching context, the effectiveness of teaching practices and, significantly, themselves as teachers” (p. 91). Psychologically speaking, emotions are intricate states that include three elements: a subjective perception, a physiological reaction, and a behavioral or expressive reaction. These responses are typically regarded as innate reactions to stimuli such as happiness, wrath, fear, and sadness. Meanwhile, feelings refer to the individual's subjective experiences of emotions or their personal interpretation and internalization of emotions. For instance, the emotion of fear can elicit sensations of uneasiness or apprehension. Similarly, the emotion of happiness can evoke sensations of contentment, joy, or exhilaration, which vary depending on the person and the situation.

One context that impacted teachers’ emotions and professional identities was the COVID-19 crisis, which transformed the educational landscape ([10]). The changes included the accelerated adoption of remote learning, redefinition of the traditional classroom setting, and an increased reliance on digital tools (e.g., Zoom, Google Meet, Microsoft Teams, and social media platforms like TikTok ([11,12]). During the pandemic, TikTok stood out for its distinctive format, emphasizing concise yet expressive storytelling through short-form videos ([13,14]). Teachers were provided a forum to openly articulate and discuss multifaceted issues, including their experiences with burnout, stress, and the complexities of adapting to remote or hybrid teaching modalities.

Although previous studies have explored TPI in TESOL, little research has examined teacher emotions as a crucial component of TPI, particularly emotions expressed in the digital spaces teachers share with their students and people outside their professional networks. Drawing upon the hypothesis that emotional vulnerability shapes TPI, this study explores the emotions and motivations of TESOL teachers who produced content for digital platforms such as TikTok. It also aims to determine how TikTok allows teachers to express their emotions and how this shapes their identities. We argue that finding a new platform to discuss emotional vulnerability promotes the (re)construction of TPI.

## 2 Literature review

### 2.1 Teacher professional identity

Teacher identity is crucial because it provides a foundation for individuals' beliefs about how to perform and understand the societal role of a teacher ([15]). Moreover, the various challenges teachers experience affect how they perceive their professional identities [16]. (2018) has suggested that identity as a teacher can be claimed, assigned, supported, or contested through individual agency and external sources. This implies that the components and indicators of this identity – what a teacher says and does – are constantly negotiated with oneself and others [17]. (2022) has stated that TPI frames teachers’ awareness of their professional roles, academic modifications to curricula, the integration of classroom techniques and strategies, and their connection to other issues in the academic context. The ways teachers perceive themselves are thus mirrored in how they exist holistically in both the classroom and the community.

As 21st-century educational methodologies began to emphasise computer-mediated education as a response to the emergence of educational technology and pedagogical innovations, there were notable shifts in TPI. The subsequent COVID-19 pandemic brought online learning to the forefront, influencing how educational institutions were run and the interactions of teachers and students. As reported by previous studies ([18,19]), many teachers experienced difficulties handling their responsibilities in a context where the rules and expectations differed significantly from face-to-face settings. In addition, teachers’ workloads increased considerably due to the challenges of converting course materials to digital formats, engaging students in the learning process, and new conceptions of "office hours" ([20,21]).

Consequently, teachers become vulnerable when long-held ideas and practices are put into question by modern educational frameworks, impacting their professional identities and credentials [22]. (2020) contends that “teachers have to reposition themselves as active agents in knowledge practices to nurture teacher digital identity and enhance students’ capacity in learning to learn” (p. 98). Moreover, since TPI impacts the capacity to exercise agency, autonomy, and career advancement, understanding how the environment affects TPI is essential to resolving issues caused by policy changes or new expectations.

One recent study on the topic was conducted by Ref. [18] (2022) in the United Arab Emirates. Focusing on the impact of the pandemic, she interviewed four higher education faculty members. Their accounts revealed that their professional identities underwent some phases of instability due to changes in their view of themselves, beliefs, and practices in the online environment. Furthermore, they experienced difficulties due to changes in their pedagogical, managerial, and social roles. In addition, switching from in-person to online instruction caused an imbalance between their personal and professional lives.

Another study that considered TPI in relation to online instruction was conducted by Ref. [21] (2021). Their study examined how three seasoned TESOL teachers who switched from in-person to online instruction handled their professional identities. The findings revealed that although all of their daily routines, knowledge, and abilities experienced reconstructions, their teaching beliefs underwent the most drastic changes. Additionally, the study concluded that alterations to a teacher's habits, knowledge, and skills result in belief negotiation and affect their professional identity. This study and others (e.g., Refs. [10,11,23]) suggest that the rapid transition to online instruction left teachers stressed, anxious, exhausted, and pressured.

### 2.2 Emotional vulnerability

Teaching is an everyday practice that involves vulnerability ([4]) and requires practitioners to embrace inevitable transitions. Teachers define and understand their professional responsibilities by considering both institutional rules and societal norms. In general, teachers experience emotional vulnerability when they fail to meet expectations, are perceived as ineffective, or face changes to the curriculum or policies ([24]). This often propels them to initiate change. Thus, vulnerability underlies teachers’ complex and multifaceted emotional experiences and is an inherent part of the profession ([4]). The structural circumstances of teaching can also reinforce vulnerability because moral and professional judgments affect how teachers perceive themselves and their competence. In addition, vulnerability serves as a strong motivator for human growth, a criterion for competence that depends on the real risk of failure.

Teachers who are reflective and sensitive in the classroom “allow students to breathe, be curious, and to explore the world and be who they are without suffocation” ([25], p. 13). They can foster true relationships with their students and create effective learning opportunities. Therefore, teachers must be willing to endure struggles to create compelling learning experiences, exercising both vulnerability and courage.

However, it is crucial to recognize the concept of 'hidden victimization' in understanding the struggles and vulnerabilities of teachers. Hidden victimization refers to the unseen or unacknowledged harm and distress teachers endure, which is not openly recognized by society, institutions, or even by the teachers themselves. This can include unrecognized stress, emotional labor, institutional neglect, and societal expectations that mask the true extent of teachers' struggles. These hidden forms of victimization can exacerbate feelings of isolation and burnout, making it essential to bring them to light to address teachers' well-being effectively.

Various studies ([4,[26], [27], [28], [29]]) have revealed that teachers become more vulnerable when pedagogical circumstances and contexts change (e.g., a shift from face-to-face to remote learning). They must drastically adjust their usual perceptions, practices, mentalities, and senses of professional identity. For example [26], (2022) interviewed 19 experienced lecturers from two Danish universities to elicit their narratives, preferences, perceptions of their roles, and senses of self to determine how the pandemic affected their professional identities. The results showed that embodied teacher identities – internalised practices transformed into dispositions – constituted a fundamental pedagogical condition and resource. The researchers also found that the sudden change resulted in a loss of TPI, concluding that the construct is sensitive to structural changes.

Similar forms of vulnerability were also evident in a large-scale cross-sectional survey conducted by Ref. [27] (2022) in northern Italy, which included 3251 teaching staff members. The researchers found that teachers showed a higher burden for emotional distress during the pandemic than the general population [29]. (2022) conducted cross-sectional empirical research with 9058 teachers in Argentina who completed self-report measures. The results revealed that over 60 % of the educators had high and moderately high stress levels related to the pandemic, work overload, and unsuitable teaching environments. Additionally, higher stress loads and psychophysical symptoms of discomfort impacted teachers’ effectiveness and sense of self, making them more likely to experience burnout.

### 2.3 TikTok: A new world for teachers

With the advent of the COVID-19 pandemic, social media use increased. The growth of the social media platform TikTok, a prominent video-sharing social networking service popular among adolescents, was particularly noteworthy ([13]) [14]. (2021) showed that TikTok gave young audiences around the globe access to a new type of short educational video. The researcher investigated how young people communicated their online learning experiences during the pandemic based on a large-scale thematic analysis of 1930 TikTok videos posted between March and June 2020. The results showed that young people saw online education as demanding and burdensome. They asked teachers for assistance, empathy, and sincerity, as well as exchanging emotional and educational support with their peers. The researcher also noted that home environments were particularly important, highlighting the connections between young people's homes, school, and social life. The study concluded that it is necessary to develop a grounded, bottom-up understanding of students' experiences and perceptions, as they choose to share them, to create a more equitable and powerful future for education.

As this study suggests, social media, especially youth-focused platforms like TikTok, can offer a valuable window into young people's experiences, including insights into their perceptions of online learning. This provides an opportunity for teachers to create more equitable and empowering educational opportunities. Additionally, TikTok can empower teachers by providing them with a platform unconnected to their institutions, which can be engaging and accessible from anywhere.

In a recent study involving two participants, one in-service and one pre-service teacher in Australia [13], (2022) explored how they engaged with TikTok content in a way related to their teacher identities, professional practices, and cultures. Using interviews, the study found that TikTok can demonstrate sides of the teaching profession (e.g., funny and entertaining) that may often be overlooked. As TikTok can shape teachers’ professional practices, the researchers recommended considering the impact of these practices on students.

All of these studies demonstrate the necessity of delving into teachers' emotional vulnerability as they perform their professional roles, as this can impact their proficiency and efficiency and push them to adapt. However, limited research exists on understanding how teachers have used digital platforms like TikTok to express their emotions and its impact on TPI.

Therefore, it is vital to investigate how teachers use TikTok to communicate their emotional vulnerability, as understanding these dynamics can provide insights into the evolving nature of teachers’ roles, the influence of technology on emotional expression, and teacher well-being in contemporary education more broadly. In this paper, we define vulnerability in a conceptual sense as the inherent potential for harm or loss, encompassing physical, emotional, and psychological susceptibilities, which can lead to growth and resilience when navigated properly. Discursively, vulnerability is constructed and perceived through language and social interactions, shaping and being shaped by societal norms, power relations, and cultural values. Here, emotional vulnerability involves acknowledging and expressing emotional susceptibilities, influenced by both personal experiences and the ways these are communicated and interpreted within social contexts.

With this dual perspective, the present study aims to explore teachers' perceptions of disclosing emotional vulnerability on TikTok and how this contributes to the ongoing refinement and evolution of their TPI. As previously discussed, emotional vulnerability can influence teachers’ practices, perceptions, and professional identities. Guided by the existing research, the present study aims to answer the following questions.

1. What are TESOL teachers' perceptions of expressing emotional vulnerability on TikTok?
2. How does expressing emotional vulnerability on TikTok affect their TPI?

## 3 Methodology

### 3.1 Research design

This study adopted an exploratory, descriptive qualitative research approach (e.g. Ref. [30]) to explore the emotional vulnerabilities of language teachers as expressed on TikTok. The primary goal was to understand how teachers use this platform to articulate, share, and manage the emotional challenges of their profession. Qualitative descriptive research was particularly suited to this context due to the complex and subjective nature of emotions, which are difficult to quantify or generalize ([31]). We aimed to reveal the underlying motivations, benefits, challenges, and implications of using TikTok to express emotions, thereby providing a deeper understanding of how digital platforms influence teachers’ professional identities.

### 3.2 Participants

This study included five secondary school teachers from Thailand. They were recruited from a Facebook group for TESOL teachers in the country. They met the following criteria: current employment as language teachers, awareness of teaching-related emotional vulnerabilities, active TikTok users since the onset of the pandemic from 2020 to the present or for at least three years (i.e., they engage with TikTok by posting, sharing, or interacting with content on the platform at least one post every week), and creators of content that addresses or reflects on their emotional challenges and vulnerabilities related to their teaching experiences.

The participants comprised four male and one female teacher, aged 24–43. They had one to nine years of teaching experience and educational qualifications ranging from BA to MA degrees (see Table 1). All participants were Thai nationals employed as secondary school teachers. To protect their identities, they were given non-identifying codes to maintain confidentiality (i.e. Participant 1, Participant 2, etc.). The Human Research Ethics Committee of 10.13039/501100010034Walailak University (WUEC-23-049-01) in Thailand granted ethical approval for the study. All participants were informed in writing about their rights as participants, the purpose of the study, and the procedure. All provided active consent.

**Table 1:** Demographic and professional characteristics.

# Non-identifying code	Gender	Age	Education	Teaching Experience
Participant 1	M	24	BA	1
Participant 2	M	34	MA	9
Participant 3	M	27	MA	4
Participant 4	M	32	BA	7
Participant 5	F	30	BA	6

### 3.3 TikTok profiles of the participants

Participant 1 shares practical tips and strategies for learning English, ranging from vocabulary explanations to grammar lessons and effective language learning techniques. He also shares his travel experiences, places, and food. He goes beyond textbook examples by demonstrating how English is used in real-life contexts, offering practical conversations, phrases, and expressions that students can readily apply. He has 2008 followers.

With 5092 followers, Participant 2 shares uplifting content on TikTok, featuring motivational messages, celebrations, and light-hearted humor. His content creates a supportive community for viewers to find inspiration and connection. He actively interacts with his audience through comments and messages, promoting meaningful connections with students, colleagues, and other followers.

Beyond educational content, Participant 3, with 1698 followers, integrates humor and relatability into his TikTok presence by creating content that addresses cultural topics, language learning challenges, and student interests, like the latest dance steps, song stitches, and others, fostering a sense of community among his followers.

Participant 4 also shares product reviews, new coffee shops in the area, travel, inspiring quotes, and daily activities on his TikTok feeds. He has 1906 followers.

Participant 5, who has 1350 followers, uses TikTok as a creative outlet to showcase her personality and personal life as a TESOL educator. She usually creates visually appealing content, often experimenting with makeup and outfits. Her TikTok feed reflects her relaxed and expressive side, featuring educational videos, playful skits, and behind-the-scenes glimpses. She shares practical teaching tips, language learning strategies, and cultural topics in a dynamic manner, fostering a sense of connection and community among language education enthusiasts.

### 3.4 Data collection

Data were collected through individual in-depth interviews conducted via Zoom, which was chosen because it creates a secure and stable environment conducive to discussing sensitive topics ([32]). Each interview lasted 50–60 min, was audio-recorded, and was transcribed. The interview guide included sections on demographic background and TikTok experience. The questions asked about the participants’ motivations, experiences with content creation, and perceptions of its impact on their professional identity.

### 3.5 Data analysis

The data were subjected to manual iterative thematic analysis following the process described by Ref. [33] (2006). This approach aligns with the exploratory, descriptive and qualitative nature of the study ([30]).

First, the recordings were transcribed and shared with participants for a preliminary member check ([34]). Then, the three researchers carefully reviewed and re-examined the transcripts to familiarise themselves with the data. Each researcher coded the transcripts independently, generating initial codes that were shared, discussed, and refined using Google Docs. They reached a consensus on the final themes and sub-themes. A code–recode strategy was implemented to enhance the consistency and dependability of the findings ([35]). All three authors revisited the data and re-coded it two weeks after the initial coding. The near-identical outcomes confirmed the integrity of the findings.

Finally, the researchers selected extracts to illustrate the themes and ensure that the analysis represented the participants’ voices. Throughout the process, the researchers maintained close communication: checking their understanding, asking for clarification and discussing any differences in their interpretation of the data. A second member check was performed to further improve the reliability of the data: participants were provided with a summary of the findings, including the themes identified and representative quotations ([34]). No participants suggested or requested any amendments in either member check.

## 4 Findings

### 4.1 TikTok as an emotional outlet

The interviews revealed that TikTok can serve as an emotional outlet for TESOL teachers, especially those who are under work-related stress. Participant 1 described the challenges of his working environment:

‘It's stressful for me. Plus, I have to handle all sorts of attitudes from students, including those from different nationalities.’

He also detailed how TikTok provides an escape:

When I'm on TikTok, I get to be someone else. Making TikTok videos lets me express myself—I dress up, put on makeup, and use filters to look great. It really helps me forget the pressures I face at work.

Participant 3 shared a similar sentiment, highlighting the communal aspect of the platform:

Sharing my emotional vulnerabilities on TikTok feels like a digital therapy session. It's become a safe space for me, almost like a digital confessional where I can share my insecurities, struggles and even my triumphs.

Moreover, Participant 3 explained that expressing his emotional vulnerability on TikTok is like a digital mural of authenticity. It is not only about sharing struggles but also about creating a raw, unfiltered narrative that challenges the conventional image of teachers as invulnerable. He recalled:

‘I'm not just a teacher in the classroom; I'm also a person with feelings. TikTok lets me create a space to recognize and support each other's emotional journeys.’

These excerpts suggest that TikTok provides a supportive environment in which TESOL teachers can express their emotions and connect with others who understand their experiences. This promotes well-being and enriches their classroom interactions.

### 4.2 TikTok as a tool for social connection

The participants also perceived TikTok as an easy way to connect with their colleagues and students. They felt that the platform makes it easier for students to open up to them, as they see their teachers as fellow TikTok users. Participant 1 shared:

‘I think TikTok can be useful for teachers too. It helps me connect with my students because now we have something in common.’

Participant 3 found that being on TikTok has helped him address student misconceptions and avoid the stigma of being strict or unapproachable. In one instance, Participant 3 narrated that one student came up to him and told him that he looked like a strict teacher in the class, but on TikTok, he was more like a student, also dancing and singing for content. Participant 3 thought that through TikTok, he could connect to his students.

Furthermore, he emphasized the importance of understanding students’ interests and using TikTok to connect with them personally.

Participant 5 has also used TikTok to encourage her students and inspire them to learn more about English. She shared:

I've connected with my students and built a good rapport with them. I feel like I'm contributing to their learning. It's not just about vocabulary but also about how language is used in real life, like giving encouragement and staying positive. I use TikTok as a way to inspire.

She also stressed that connections can enhance understanding and motivation:

Building a strong connection with my students is crucial. I work hard to create a positive relationship with them because it's tougher to teach effectively if we're not close. They understand me and the lessons better when there's a good connection.

These findings highlight the potential of TikTok to foster positive connections between TESOL teachers and their students, enhancing understanding, motivation and engagement in the learning process.

### 4.3 Teachers’ TikTok identities

The study also revealed that TESOL teachers adopt distinct identities on TikTok that differ from their classroom personas. On TikTok, they become more expressive, emotional, and creative. Participant 1 explained:

In the classroom, I always dress formally and act the part of a teacher. I'm careful with what I say and do because I need to keep it professional. But on TikTok, I can show a different side of myself.

However, participants mainly expressed positive emotions on TikTok, aware that their students would see what they posted. Participant 2 stated:

‘I try not to share any sad or tough feelings on social media, especially TikTok, since I know some of my students follow me there.’ This highlights TESOL teachers' conscious efforts to maintain a positive image on the platform.

Despite the myriad advantages of TikTok, the participants also acknowledged some negative experiences. They reported being sensitive to the content, comments and reactions they have received from other users. For instance, Participant 5 narrated:

In one of my dancing TikTok content, one of my followers said that I don’t know how to dance, and that I should just do make-up tutorial or product review instead of dancing on TikTok.

When asked what she did with the comments and the follower, she said that she blocked her follower and deleted the comment. She mentioned further:

I was hurt but it’s my life and I enjoyed dancing on TikTok so I avoided those negativities.

Participant 3 noted:

‘Even when you know what could happen when you post something on TikTok, the comments from others can still get to you. We're all human, after all.’

To cope with this vulnerability, participants have tried to avoid focusing on negative comments and concentrate on the positive aspects of their experience. Participant 3 shared:

Sometimes, I just ignore what other people say on TikTok, especially if they're being negative. I either unfollow them or, most of the time, I just block them. You know, if you let that negativity get to you, it can really affect your teaching.

Overall, the findings suggest that TikTok provides a platform for TESOL teachers to express their emotions, share relatable content and amplify their voices. As Participant 5 stated:

TikTok is more than just a place for the latest trends and challenges. For me, as a teacher, it's like a digital revolution for how I feel. With TikTok, I can change the way I talk about teaching. It lets me focus more on teachers' emotions and make the whole experience more well-rounded.

Thus, TikTok not only enables teachers to express themselves, enhance understanding, and improve classroom interactions, but it also develops connectivity, influences instructional methods, and broadens outreach capacities, allowing teachers to exchange resources and experiences ([15]).

These findings contribute to a deeper understanding of how TikTok shapes TESOL teachers’ emotional experiences and social interactions. By providing an emotional outlet, fostering connections, and allowing teachers to develop unique identities, TikTok has the potential to transform the way they engage with their profession and their students. The findings have significant implications for education, highlighting the need to consider the role of social media platforms in shaping teacher-student relationships and emotional well-being.

## 5 Discussion

### 5.1 TikTok as an emotional outlet

Our findings highlight the role of TikTok as an emotional outlet for 10.13039/100026623TESOL teachers, consistent with prior research emphasizing the importance of emotional support in the profession ([36]). The participants reported using TikTok to cope with stressors, such as problematic student behavior and excessive workloads, in technology-mediated teaching and learning environments (e.g., Refs. [20,21]). The teachers use TikTok to engage in activities that make them feel happier and more relaxed, such as producing and sharing content, communicating with other users, and watching motivational videos. These activities allow them to escape their stressful working environments, have fun, unwind, and forget their worries.

The findings also suggest that teachers use TikTok to create a digital space for authenticity and mutual support by sharing their emotional vulnerabilities. These findings corroborate prior research findings that teachers use social platforms to manage their emotions and maintain well-being ([[37], [38], [39]]). The use of TikTok as an emotional outlet underscores the importance of addressing teachers' emotional needs in the context of their professional lives.

### 5.2 TikTok as a tool for social connection

The TESOL teachers considered TikTok to be a valuable tool for connecting with their students outside of traditional communication channels. It allows teachers to discover their students' interests and preferences, informing their pedagogical practices. The participants believed that sharing a common interest in TikTok enabled them to develop rapport with their students and cultivate a positive classroom environment, which helps students learn more effectively. Some teachers even used TikTok to encourage and inspire their students to learn more about the English language. Although some teachers post about their hobbies and favorite activities on TikTok, they still manage to inspire their students through the use of English in their content. Students can learn vocabulary and word usage from their teachers' TikTok videos by watching to these TikTok contents. This is evident in Participant 5, who not only posts trendy activities like dancing and product reviews but also demonstrates the correct use of English. This may inspire not only their students but also those who are following their TikTok accounts.

The findings highlight the potential of TikTok to foster positive connections between TESOL teachers and their students, enhancing their understanding, motivation, and engagement. For example, a teacher might create a TikTok video incorporating a popular trend or song their students enjoy and then use it as a springboard for a language learning activity. Moreover, the informal nature of TikTok interactions can help break down barriers between teachers and students, creating a more relaxed and open learning environment. This result is consistent with research emphasizing the importance of teacher-student relationships for academic achievement ([40,41]). Like other social media platforms, TikTok can enhance these relationships and create more personalized learning experiences.

### 5.3 Teachers' TikTok identities

Our study revealed that the TESOL teachers viewed TikTok as a valuable outlet that allows them to detach from their role as teachers and express themselves more freely. They can speak and dress informally or use various filters to express themselves on the platform. Although the participants acknowledged that using TikTok made them emotionally vulnerable, they described strategies for handling negative comments and maintaining professional boundaries, such as ignoring negative comments and unfollowing or blocking users.

The findings suggest that teachers can use TikTok to showcase aspects of their personalities that are less apparent in the classroom setting. For instance, a teacher might share a personal story or hobby, allowing students to see them as a multi-dimensional individual with interests and experiences outside of teaching. This self-expression can help humanize teachers and create a more authentic and relatable learning environment. However, the findings also highlight the need for teachers to navigate the emotional challenges of using social media platforms and develop strategies for maintaining professional boundaries and positive online interactions. These findings indicate that TESOL teachers can use TikTok as a platform for self-expression while remaining professional, which aligns with a previous study on teachers’ use of social media ([13]).

The EFL teachers we interviewed have cultivated distinct TikTok identities, which differ from their professional classroom identity. They view TikTok as a valuable outlet that allows them to detach themselves from their role as teachers. For instance, they can speak, dress informally, and use various filters to express themselves. The teachers expressed awareness that students follow them on TikTok, but they believe it is a positive thing, as students can understand their interests and personalities outside of the classroom and see aspects of their identities that are often hidden.

Although participants acknowledged that using TikTok made them emotionally vulnerable, they described strategies for handling negative comments and maintaining professional boundaries. Specifically, they emphasized the importance of staying positive and avoiding negative videos, comments, and reactions. The teachers are mindful of the need to appear cheerful even if they feel sad, as negativity might impact their interactions and classroom teaching. When confronted with harsh criticism in the comments on their posts, the teachers feel it is prudent to block those users and move on. These findings indicate that EFL teachers can use TikTok as a platform for self-expression while maintaining their professional identity.

### 5.4 Limitations and future research

This study has offered valuable insights into the role of TikTok in shaping the emotional experiences, social interactions, and identity development of TESOL teachers. However, it has some limitations. First, the study relied on a small sample size of TESOL teachers in Thailand, which may limit the generalisability of the findings to other contexts. Future research could explore the experiences of teachers who use TikTok in different cultural and educational settings to provide a more comprehensive understanding of the phenomenon. Second, the study relied on interview data, which may be subject to self-report and social desirability biases. Future studies could employ additional data collection methods (e.g. questionnaires and content analysis).

## 6 Conclusion and implications

Our findings contribute to an understanding of how TikTok shapes teachers' emotional experiences and social interactions. TikTok helps teachers express their emotions, develop relationships with their students, and project unique identities. However, the platform also reveals the complexity of emotional vulnerability, acting as both a cause and an effect of TikTok use. While it provides an outlet for emotional expression and support, it can also expose teachers to additional scrutiny and stress.

Institutions and policymakers should prioritize addressing the systemic causes of teacher stress and workload within neoliberal educational frameworks rather than solely focusing on coping mechanisms through social media platforms like TikTok. This approach aims to reduce the burdens imposed on teachers and promote sustainable well-being by revisiting policies and practices that contribute to excessive work demands. Efforts should be directed toward creating supportive environments that alleviate these pressures, fostering healthier professional conditions for educators. They could also provide workshops about using TikTok for self-care and emotional regulation or create an online community of practice (OCoP), a virtual space where teachers can share experiences, support each other, and collaborate on best practices for using TikTok in education. This could lead to fostering mutual learning and professional development through the exchange of knowledge and resources.

Moreover, the findings highlight that TikTok can lead to more personalized and engaging learning experiences. Institutions should explore how social media platforms, including TikTok, can foster positive connections between teachers and students while mindful of not adding to teachers' existing workload burdens. By strategically integrating these platforms into educational practices, educators can enhance student engagement and create supportive learning environments beyond the traditional classroom setting. This approach should be accompanied by supportive policies that promote responsible and effective use of social media, ultimately contributing to a balanced approach that supports both teachers' well-being and educational outcomes.

Finally, the findings suggest it is important to support teachers' self-expression and identity development. Institutions should create a supportive environment where teachers can express themselves authentically while they fulfill their professional responsibilities. This may involve providing training and resources on social media use and fostering a culture of respect and understanding for teachers' personal lives and identities. While our study primarily focused on young and relatively new TESOL teachers, we acknowledge the potential benefits of TikTok for senior educators as well. Recognizing that senior teachers may face challenges in navigating new technologies like TikTok, we propose that workshops and support mechanisms can be tailored to their needs. These initiatives could provide essential training on how to effectively use TikTok for professional development, emotional support, and fostering connections with students.

In conclusion, our study highlights the complex and multifaceted role of TikTok in shaping TESOL teachers' emotional experiences, social interactions, and identity development. The dual nature of emotional vulnerability—both as a cause and an effect of TikTok use—underscores the need for thoughtful integration of social media in educational contexts. The findings have significant implications for the future of education. By leveraging the potential of platforms like TikTok, institutions can create a more supportive, engaging, and authentic learning environment for both teachers and students. As social media continues to evolve and play an increasingly important role in education, researchers and practitioners must continue exploring how these platforms can be harnessed to support educational success.

## Availability of data and material

Data will be made available on request.

## Funding

This research has been funded by the 10.13039/501100010034Walailak University Internal Research Fund (WU66256).

## CRediT authorship contribution statement

**Henry E. Lemana II:** Writing – original draft, Funding acquisition, Conceptualization. **Mark B. Ulla:** Writing – review & editing, Writing – original draft, Methodology, Formal analysis, Conceptualization. **Lucas Kohnke:** Writing – review & editing, Writing – original draft, Formal analysis, Data curation.

## Declaration of competing interest

The authors declare that they have no known competing financial interests or personal relationships that could have appeared to influence the work reported in this paper.
